# New Facts with Regard to Strangulated Hernia

**Published:** 1841-02

**Authors:** 


*New facts with regard to Strangulated Hernia.* By M. Laugier.—Every one knows that in strangulated hernia, the upper extremity of the intestine, or that part which is comprized between the stomach and the seat of strangulation, becomes distended by fluid, gaseous, or fecal matters, whilst the lower portion remains flaccid, or, at least, the matters found in it do not distend it more than usual.

No attempt has yet been made to draw practical inferences from this fact, so as to use it as a means of distinguishing intestinal from omental hernia; or which portion of the intestinal tube, whether the large or small intestine, was contained in the hernial sac. This has, however, been done by M. Laugier, who considers it both as a simple and sure means of diagnosis, besides possessing the additional advantage of indicating the relative urgency of the case for operation. The results of his observation are embodied in the following conclusions.

1. The extent, the form, and seat of the meteorism of the belly, during strangulated hernia, vary according as the hernia consists of omentum or intestine, and according as the upper extremity consists of the small or the large intestine. Its extent varies also according as the portion of small intestine included in the hernia is more or less distant from the stomach.

2. In omental hernia, before the development of peritonitis, the belly is soft, and flat even in the neighborhood of the hernia. There is no meteorism.

3. Meteorism appears very rapidly when the hernia consists of intestine.

4. When the hernial sac contains the large intestine, the sigmoid flexure of the colon for example, the upper extremity consisting of nearly the whole of the intestinal tube, the meteorism is general, and gives to the abdomen an almost cylindrical form.

5. If the small intestine is alone in the hernial sac, or along with a portion of omentum, the flakes and the epigastric region are soft and depressed: the swelling of the abdomen occupies the hypogastric and umbilical regions. The swelling is spherical, or nearly so, resembling in this respect some encysted tumours, or the uterus at the sixth or seventh month of pregnancy, from which, however, it differs in many essential characters.

6. When the strangulated part of the intestine is near the stomach, the swelling of the abdomen is trivial, in proportion to the duration of the strangulation; for in that case the upper extremity of the tube is only of small extent.

7. From these facts may be deduced the unexpected and important conclusion, that in crural hernia, and in some inguinal hernias, where gangrene is most to be feared, all haste should be used either to reduce it by the taxis or by operation; for if by the occurrence of gangrene of the intestine an artificial anus should be formed, its proximity to the stomach renders it more dangerous for the patient. A swelling of small extent, limited to the neighborhood of the hernia generally, inspires a security which may lead to fatal results; whilst it is the general meteorism, or one, at least, which is very extensive, that alone permits of delay, since it indicates the incarceration of a portion of intestine which is far from the stomach.

8. It is only before the accession of general peritonitis that the characters of the windy swelling, above indicated, can be relied on; they are most marked at that stage.

M. Laugier adds, that these remarks have been all verified by careful clinical observation, and by the result of many operations for hernia.—*Ed. Med. and Surg. Jour.*, July, 1840. from *Comptes Rendus*, 2d March, 1840. *Ibid.*

